# Photobiomodulation Activates Coordinated Signaling Networks to Modulate Inflammation, Adaptive Stress, and Tissue Healing via Redox-Mediated NFκB–TGF-β1–ATF-4 Axis

**DOI:** 10.3390/cells15010088

**Published:** 2026-01-05

**Authors:** Sasikumar Ponnusamy, Mahmud Amin, Amruta Bhat, Sarah Garczynski, Saeed Ur Rahman, Sailee Rasam, Sharaschandra Reddy Govindool, Imran Khan, Praveen Arany

**Affiliations:** 1 Oral Biology, Surgery, and Biomedical Engineering, University at Buffalo, Buffalo, NY 14214, USA; sponnusa@buffalo.edu (S.P.); mmamin@buffalo.edu (M.A.); amrutaga@buffalo.edu (A.B.); sgar202@gmail.com (S.G.); saeedbio80@gmail.com (S.U.R.); rasamsailee1@gmail.com (S.R.); sgovindo@iu.edu (S.R.G.); 2 Medicine, Jacobs School of Medicine and Biomedical Sciences, University at Buffalo, Buffalo, NY 14214, USA; 3 Women’s Malignancies Branch, National Cancer Institute, National Institutes of Health, Bethesda, MD 20892, USA; imran.khan@nih.gov

**Keywords:** photobiomodulation, ATF-4, TGF-β1, ROS, NFκB, LLLT

## Abstract

**Highlights:**

**What are the main findings?**

- Photobiomodulation evokes intra- and extracellular redox mechanisms.
- NFκB coordinates PBM-activated TGF-β–ATF-4 signaling.

**What are the implications of the main findings?**

- Anti-inflammatory clinical responses to PBM therapy can be better rationalized.
- Framework for integration of adaptive stress and tissue healing responses by PBM.

**Abstract:**

Photobiomodulation (PBM) therapy has been effectively used to relieve pain and inflammation and promote tissue healing and regeneration in a broad range of ailments. Prior work has focused on intracellular mitochondrial cytochrome c oxidase, while extracellular latent TGF-β1 activation had been noted. This work investigated the role of PBM-generated redox signaling and integration in normal oral keratinocytes, using Western blots and pathway-specific small molecule inhibitors. We observed that PBM primarily generates ROS intracellularly within mitochondria, which then diffuse extracellularly to activate latent TGF-β1. This activation triggers ATF-4 expression through both canonical (Smad3) and non-canonical (p38, ERK) TGF-β signaling pathways. We observed a critical role for NFκB as an essential integrator, coordinating these responses as evidenced by the loss of ATF-4 expression following NFκB inhibition (BAY II) after both PBM and TGF-β1 treatments. Proteomic pathway analysis revealed that PBM downregulates inflammatory and apoptotic pathways while activating stress-adaptive responses in the NFκB pathway. A core set of PBM-induced redox, NFκB, and TGF-β signaling targets was identified. These findings suggest that optimal PBM treatment responses require a coordinated action of multiple signaling pathways that optimize cellular adaptation to stress and promote tissue repair rather than protracted inflammation and cell death.

## 1. Introduction

Photobiomodulation (PBM) or low-level light/laser therapy (LLLT) utilizes non-invasive, low-dose light in the visible or near-infrared wavelength to modulate (inhibit or stimulate) various biological responses for clinical therapeutic benefits [1,2]. PBM treatments have been used for six decades as a clinical treatment for a broad range of ailments with evidence from case reports, lab studies, and rigorous recent clinical trials [3,4,5,6,7,8,9,10,11,12]. Among them, effects on epithelial wound healing and oral mucositis have been at the forefront, with rigorous systematic reviews and meta-analyses leading to clinical practice guidelines [13,14,15,16]. Given the wide array of clinical applications, the precise molecular mechanisms operative in specific clinical scenarios remain unclear, adding uncertainty to clinical dosing rationale and generating inconsistent therapeutic outcomes.

A majority of the mechanistic PBM studies have focused on the role of cytochrome c oxidase in the mitochondria, which has been noted to be capable of absorbing light and transiently increasing its role in electron transport, generating ROS and ATP [17,18]. In contrast to this intracellular mechanism, PBM treatments have been noted to activate extracellular latent TGF-β1, a potent multifaceted growth factor capable of a broad range of therapeutic benefits [19,20,21]. This photoactivation process involves a redox-sensitive oxidizable amino acid, methionine, at position 253 on the latency associated peptide of TGF-β1 that involves a conformational change, releasing the active dimer. The presence of this latent growth factor outside the cell has raised interesting questions about the source and target of PBM-induced redox generation.

A key question in the PBM field is clinical dosing, especially since clinical treatments are subtle and barely perceptible to either subject or operator. This has prevented the development of clear guidelines for maximal dosing and optimal benefits [22,23,24]. To address this critical issue, dose escalation animal studies were performed that outlined the role of increased surface temperature (>45 °C) and the downregulation of a phototoxicity-specific endoplasmic reticulum stress pathway target, Activating Transcription Factor-4 (ATF-4) [25]. This study examined the ability of ATF-4 to specifically detect phototoxic stress that, in turn, modulates the integrated stress response and heat shock responses to escalating PBM dosing. As PBM is a low-dose phenomenon, the upper limit of the dose threshold has been a primary concern, represented by phototoxic stress responses in the endoplasmic reticulum mediated by selective translation (via the internal ribosome entry site on the promoter) of ATF-4 that has a central role in inducing gene expression in antioxidant responses, amino acid metabolism, autophagy, apoptosis, and unfolded/heat shock protein responses [26,27,28,29]. The coordinated ATF-4 response determines whether PBM could induce repair and resolution via autophagy and reconstitution at optimal doses or induce a combination of apoptosis and autophagy at excessive doses. Using a combination of in vitro and in vivo mouse models, this study outlines the correlation between increasing ATF-4 expression and therapeutic PBM dosing, whereas ATF-4 downregulation leads to excessive autophagy and concurrent initiation of apoptosis, indicating that the maximal PBM dose threshold has been exceeded. Thus, this suggests that ATF-4 expression can be used as a molecular biomarker in tissue biopsies or biofluids collected post treatment and analyzed using immunoassays (e.g., ELISA or Western blots) to verify maximal clinical safety and therapeutic efficacy with PBM treatments. However, the signaling pathways mediating PBM-induced ATF-4 expression remain to be elucidated.

PBM treatments have been noted to mitigate inflammation in a broad range of ailments in response to various damage- and pathogen-associated molecular patterns [30,31]. The roles of TNFα and NFκB signaling in mediating inflammation and affecting tissue healing and regeneration have been well established [32]. PBM activation of NFκB signaling has been noted to modulate the inflammatory response [33]. Other studies have observed the ability of PBM treatments to induce NFκB-mediated gene expression through retrograde mitochondrial signaling by modulating mitochondrial membrane potential and ROS generation [34]. The role of reverse mitochondria in nuclear (retrograde) signaling appears to have a prominent effect on genome integrity, dynamics, or turnover, impairing the oxidative phosphorylation machinery, activating the mitochondrial integrated stress response, eliciting chromatin remodeling, and promoting cellular immaturity rather than apoptosis to yield metabolic dysfunction [35]. Retrograde signaling can be mediated indirectly via metabolic cues or directly by changes in intracellular calcium dynamics that result in changes in gene expression. These are distinct from the mitochondrial calcium release accompanied by cytochrome c and other proapoptotic factors that lead to cell apoptosis. A major outcome of retrograde signaling is a change in stress-related pathways that serve as an adaptive response to improve cell resilience and reestablish physiological cellular homeostasis.

The dichotomous role of inflammation in physiological homeostasis, especially tissue healing, has been shown to determine the ultimate biological outcome [36,37,38]. Unresolved or low amounts of persistent inflammation leading to oxidative damage have been noted to contribute to wound chronicity [39,40]. Interestingly, a salient clinical observation is the reduced healing capacity with aging [41,42]. This has provided new strategies to improve tissue healing by targeting senescent cells [43]. The elucidation of the senescence-associated secretory phenotype (SASP) involving key inflammatory cytokines suggests that it contributes to one of the putative mechanisms of cellular senescence that has been termed inflammaging [44,45]. Thus, resolving inflammation appears to be a key aspect of the initiation of repair and contributes to mitigating age-related deterioration.

The present study was motivated by these gaps in knowledge and aimed at investigating the roles of PBM-generated redox modulation of the ATF-4 and TGF-β1 pathways in normal oral keratinocytes cells. Cells were treated with PBM treatments in a dose-escalating manner and analyzed for discrete signal transduction pathways using Western blots and a proteomic array.

## 2. Materials and Methods

### 2.1. Cell Lines

Human normal oral keratinocytes (NOKSI) were provided by Dr. Silvio Guhkind, while NIDCR/NIH and human oral fibroblasts (HOFs) and human cortical neurons (HCNs) were procured from ATCC [46]. These cell lines were maintained in Dulbecco’s Modified Eagle Medium (GE Healthcare Life Sciences, Malborough, MA, USA) supplemented with 10% fetal bovine serum (Atlas Biologicals, Fort Collins, CO, USA) with Penicillin and Streptomycin (ThermoFisher Scientific, Waltham, MA, USA). Cells were grown at 37 °C in an incubator with 5% CO_2_.

### 2.2. PBM Treatments

A diode laser was used (810 nm, CW, 10 mW/cm^2^, 300 s, 4.5 p.J/cm^2^, 1 ɇ, Thor Photomedicine, Amerhsam, UK to perform PBM treatments in a non-thermal manner based on prior studies in the lab [21,25,47]. The distance varied according to the spot size necessary to treat a specific plate format, which was captured by the tissue surface irradiance (mW/cm^2^) assessed before every study with an optical power meter (PM200, Thor Labs, Newton, NJ, USA) that combined the power output, spot size, and distance [48]. The addition of individual wavelength photon energy (eV) is reported as the photon fluence (p.J/cm^2^) adjusted to the standard dose termed Einstein (ɇ), as described previously [49,50].

### 2.3. ROS Detection in Extra- and Intracellular Milieu

To assess PBM-induced ROS generation, two chemiluminescence assays were performed with luminol (detects both extra- and intracellular ROS) and isoluminol (extracellular ROS only) [51]. The solutions were freshly prepared by mixing 5 mg of luminol powder with 100 mL of 1 M sodium hydroxide (both Sigma-Aldrich, St. Louis, MO, USA), mixed gently, and added immediately to the wells (1 mL/well (*v*/*v*)) after laser treatments, and chemiluminescence was detected using a microplate reader (MiniMax 3, Molecular Device, San Jose, CA, USA). Similarly, isoluminol (0.2 mg/mL, 0.1 mM) was prepared and used in these studies to detect extracellular ROS. To examine the role of ROS, two scavengers, namely, N-Acetylcysteine (NAC, 10 μM, both extra- and intracellular) and Catalase (CAT, 100 μM, extracellular only) were pretreated 30 min prior to PBM treatments.

### 2.4. TGF-β1 Treatments

To examine the role of TGF-β1 signaling, recombinant TGF-β1 (2.5 ng/mL, R&D Systems, Minneapolis, MN, USA) was used in 0.2% FBS-DMEM. Cells were preconditioned for up to 2 h in low serum to reduce background TGF-β levels prior to beginning these studies [52,53,54].

### 2.5. Pathway-Specific Inhibitors

To examine the signaling pathways induced by PBM, pretreatment with signaling inhibitors of TGF-βR1 inhibitor (SB431542, 10 μM, Calbiochem, San Diego, CA, USA), Smad (SIS3, 10 μM, Calbiochem, USA), p38 (SB202190, 10 μM, Calbiochem, USA), ERK/MAPK (PD98059, 25 μM, Calbiochem, USA), NF-κB (BAY 11-7082, 10 μM, Calbiochem, USA), PI3K (LY 294002, 10 μM, Calbiochem, USA), and JNK (JNK inhibitor II, 10 μM, Calbiochem, USA) were used 30 min prior to PBM treatments.

### 2.6. Western Blotting

The cells were lysed in RIPA buffer with Complete-Mini Protease inhibitors (both Sigma-Aldrich, St. Louis, MO, USA), sonicated briefly (QSonica, Newton, CT, USA), and centrifuged at 14,000 rpm (23,790 *g*) at 4 °C for 10 min. Total protein was assessed with a Bradford assay kit (BCA Protein Assay, Thermo Scientific Inc., Waltham, MA, USA). Then, protein lysates were separated in precast gels and transferred onto PVDF membranes (both Bio Rad, Hercules, CA, USA), blocked with 1% BSA for 1 h, and incubated with the primary antibodies ATF-4 (1:1000, Abcam, Cambridge, MA, USA), Phospho-Smad 2 and 3 (1:1000, Cell signaling), or β-actin (1:30,000, Cell signaling) at 4 °C overnight. The following day, after washing, the blots were incubated with appropriate species-specific HRP-conjugated secondary antibodies (Cell signaling, Danvers, MA, USA), developed using chemiluminescent substrates (Thermo Scientific, USA), and digitally imaged (ChemiDoc, Bio-rad, USA). ATF-4 bands were quantitated using NIH ImageJ (v1.54r) and normalized to β-actin. Data is representative of at least two individual studies.

### 2.7. NFκB Pathway Array

To examine the role of NFκB signaling, a human proteome NFκB kit was used (R&D Biosystem, Minneapolis, MN, USA). Briefly, NOKSI cells were seeded (500,000/well) in seven groups and treated with PBM, TGF-β1, SB431542, BAY 11-7082, NAC, or Catalase. After 24 h, the cells were harvested and incubated with NFκB Pathway Array membrane as per the manufacturer’s instructions. Briefly, the membranes were blocked for one hour with blocking buffers, treated with the respective group cell lysate, and incubated overnight at 4 °C on a rocking platform. The next day, the array membrane was washed and incubated with a detection antibody cocktail followed by a Streptavidin–HRP conjugated secondary antibody, and chemiluminescence was detected using the ChemiDoc system (Bio Rad, USA). Images were analyzed for mean pixel density using NIH Image J (v1.54r) and heat map of the mean intensity value was generated using conditional formatting (3-color scale) in Excel (Microsoft, Seattle, WA, USA).

### 2.8. STRING-Based Protein–Protein Interaction (PPI) and Pathway Analysis

To investigate the functional relationships among the identified genes, a protein–protein interaction (PPI) network was constructed using the STRING database (v12.0), with the organism set to Homo sapiens and a minimum interaction confidence score of 0.5. Clustering of the PPI network was carried out using the Markov Cluster Algorithm (MCL) within STRING, with the inflation parameter set to 3, enabling the identification of functionally coherent protein interaction modules [55].

## 3. Results

### 3.1. Cellular Context for PBM Generation of ROS

**Figure 1:**
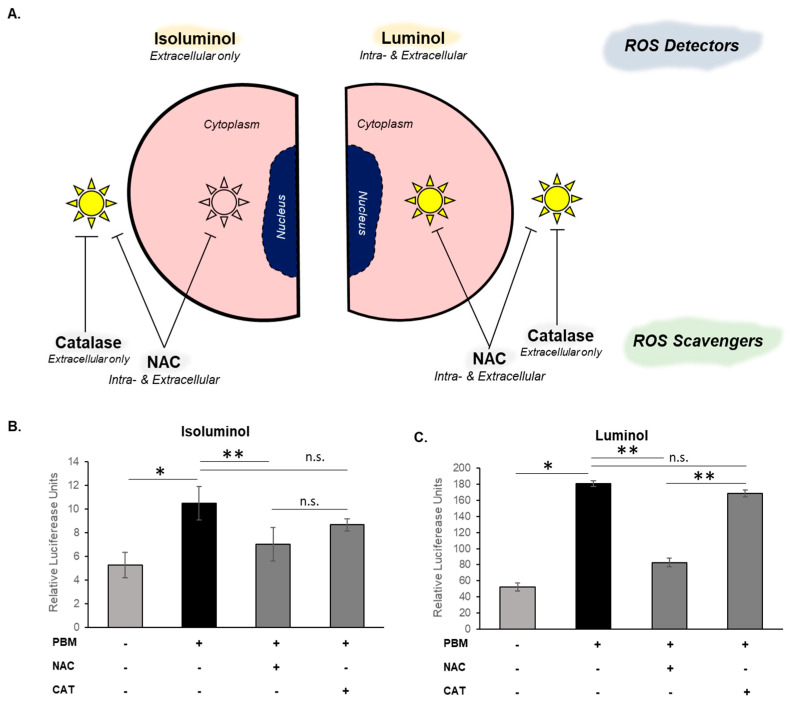
Cellular context for PBM-induced ROS generation. (**A**) Outline of the experimental setup using chemiluminescent assays and ROS scavengers with PBM treatment in oral keratinocytes. (**B**) Isoluminol assay for extracellular ROS production following PBM treatment with presence of NAC or Catalase. (**C**) Luminol assay for intracellular ROS production following PBM treatment, with presence of N-Acetylcysteine (NAC) or Catalase. Data is presented as mean ± standard deviation where * *p* < 0.05 and ** *p* < 0.05 denotes statistical significance, n.s.—not significant.

To examine the precise cellular context of PBM-generated redox signaling, intracellular and extracellular ROS were quantified with luminol (extra- and intracellular) and isoluminol (extracellular only) (Figure 1A). Further, preincubation with either NAC (extra- and intracellular) or Catalase (extracellular only) before PBM treatments was performed on oral keratinocytes. We observed that ROS were detected in the cell milieu and were more significantly (*n* = 3, *p* < 0.05) neutralized by NAC than Catalase (Figure 1B,C). The most striking observation was the lack of significant (*n* = 3, *p* < 0.05) neutralization of the intracellular ROS with NAC detected by luminol. This suggests that the primary site of PBM-induced redox is intracellular, likely the mitochondrial cytochrome c oxidase that has been shown in several prior studies to effectively serve as a chromophore.

### 3.2. PBM Induces ATF-4 via TGF-β1

**Figure 2:**
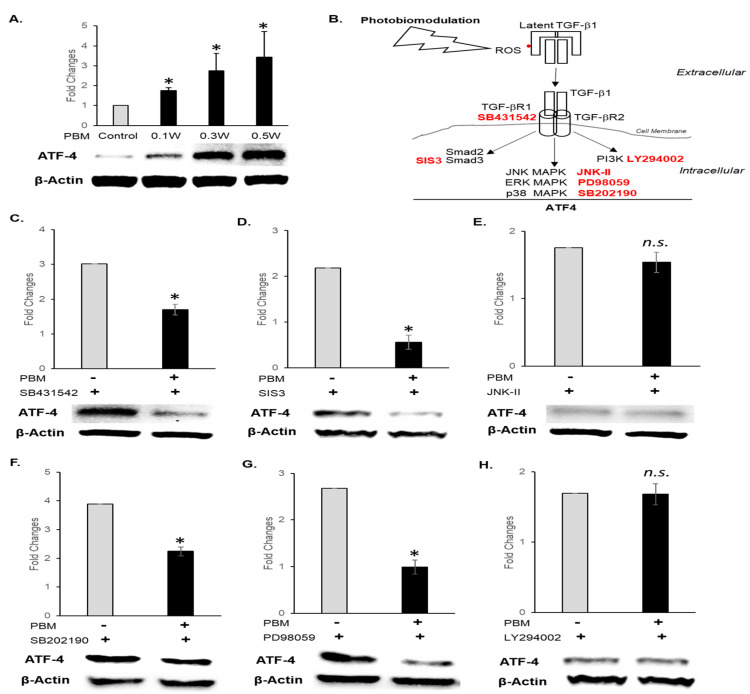
Western blots for ATF-4 expression in oral keratinocytes following PBM treatments. (**A**) ATF-4 expression after different dosages of PBM treatment. (**B**) Outline of the PBM-induced ATF-4 expression assessed following preincubation with pathway-specific inhibitors. (**C**) TGF-βR1 inhibitor (SB431542), (**D**) Smad inhibitor (SIS3), (**E**) JNK inhibitor (JNK inhibitor II), (**F**) p38 inhibitor (SB202190), (**G**) ERK/MAPK inhibitor (PD98059), (**H**) PI3k inhibitor (LY 294002). Normalized band quantitation is presented as mean ± standard deviation where * *p* < 0.05 denotes statistical significance, n.s.—not significant.

We first performed a PBM dose (0.1, 0.3, and 0.5 W, 1 to 10 mW/cm^2^ for 300 s, 0.45 to 4.5 p.J/cm^2^, 0.1 to 1 ɇ) escalation study and noted that ATF-4 expression progressively increased, correlating with a proliferative response (Figure 2A and Supplementary Figure S1A). We also examined cells of other lineages, namely, human oral fibroblasts and human cortical neurons, and noted that ATF-4 expression and cell survival varied with PBM treatments at various doses (Supplementary Figure S1B,C). Based on these observations, further signaling studies in keratinocytes were performed at 10 mW/cm^2^, 300 s, 4.5 p.J/cm^2^, and 1 ɇ.

We had previously observed that extracellular latent TGF-β1 is activated by PBM treatments via a redox mechanism, while ATF-4 expression is induced in the endoplasmic reticulum intracellularly [20,25]. We next examined whether these two observations could be linked. We performed a loss-of-function small molecule inhibitor screening strategy to dissect both canonical and non-canonical TGF-β signaling. Preincubation with TGF-β Receptor (Alk-5) inhibitor (SB431542), Smad 3 inhibitor (SIS3), ERK/MAPK (PD98059), p38 (SB202190), JNK (JNK Inhibitor II), and PI3K (LY294002) was performed prior to PBM treatments. We observed that inhibiting TGF-β signaling via Alk5, Smad3, p38, and ERK significantly (*p* < 0.05) abrogated PBM-induced ATF-4 expression (Figure 2C,D,F,G). However, the blocking of JNK and PI3K showed no significant differences from PBM treatments alone (Figure 2E,H). These results indicate that PBM treatments induce ATF-4 expression via concerted TGF-β1 signaling involving the Alk5, Smad3, ERK, and p38 MAPK pathways.

### 3.3. TGF-β1 Signaling Induces ATF-4

**Figure 3:**
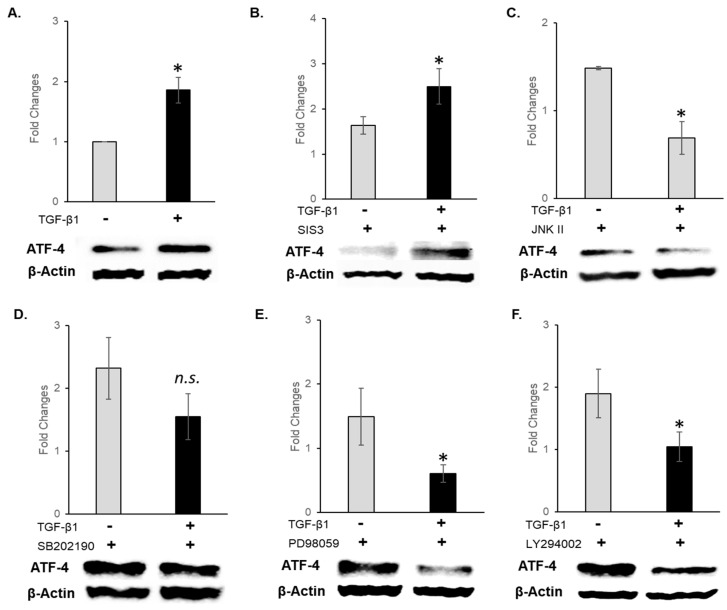
Western blots for ATF-4 expression in oral keratinocytes following TGF-β1 treatments. (**A**) ATF-4 expression after recombinant TGF-β1 treatment. Preincubation with pathway-specific inhibitors prior to TGF-β1 treatments was performed. (**B**) Smad inhibitor (SIS3), (**C**) JNK inhibitor (JNK inhibitor II), (**D**) p38 inhibitor (SB202190), (**E**) ERK/MAPK inhibitor (PD98059), (**F**) PI3K inhibitor (LY 294002). Normalized band quantitation is presented as mean + standard deviation where * *p* < 0.05 denotes statistical significance, n.s.—not significant.

To further validate this observation, we directly examined the role of TGF-β1 signaling on ATF-4 expression. Recombinant TGF-β1 treatments significantly (*p* < 0.05) upregulated ATF-4 expression compared to the control (Figure 3A). Inhibiting the Smad (SIS3) pathway, surprisingly, did not affect upregulated ATF-4 expression. However, all other signaling pathway inhibitors, namely, JNK, ERK, and PI3K, significantly (*p* < 0.05) downregulated TGF-β1-induced ATF-4 expression (Figure 3B,C,E,F).

While p38 inhibition showed a trend toward reduction, it did not appear to be statistically significant (Figure 3D). These results indicate that while TGF-β1 signaling directly modulates ATF-4 expression, it appears to vary from PBM-induced prototypical TGF-β signaling, suggesting that alternative PBM signaling pathways are operative.

### 3.4. NF-κB Signaling Integrates Intracellular Redox and PBM–TGF-β-Induced ATF-4

**Figure 4:**
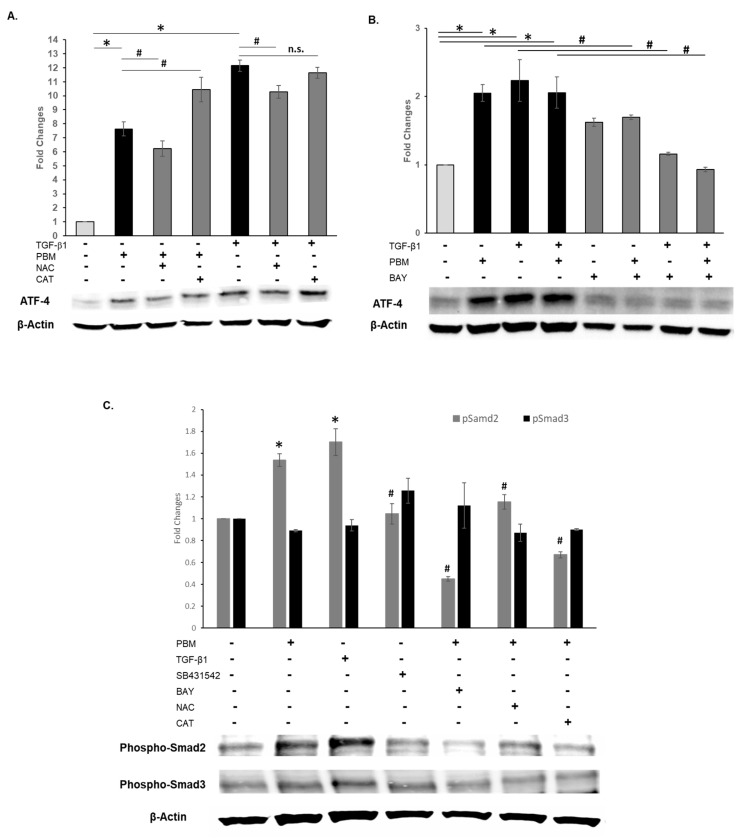
Western blots for ATF-4 and Phospho-Smad in oral keratinocytes following PBM or TGF-β1 treatments. (**A**) Preincubation with ROS scavengers, NAC, or Catalase prior to PBM or TGF-β1 treatment on ATF-4 expression. (**B**) Evaluating the role of NFκB with the inhibitor BAY-II. (**C**) Activation of TGF-β signaling was evaluated by Phospho-Smad 2 and 3 following NAC, Catalase, SB431542, and Bay-II pretreatments prior to TGF-β1 or PBM. Normalized band quantitation is presented as mean ± standard deviation, where * *p* < 0.05 compared to control or ^#^ *p* < 0.05 compared to PBM denote statistical significance, n.s.—not signficant.

Next, we examined the role of PBM-generated redox-induced signaling in modulating ATF-4 expression. Pretreatments with NAC, but not Catalase, significantly (*p* < 0.05) reduced ATF-4 expression (Figure 4A). TGF-β1 treatments more robustly induced ATF-4 expression that was also similarly significantly (*p* < 0.05) modulated by global ROS quenching with NAC, but not extracellular ROS neutralization with Catalase.

These results indicate that redox pathways are key to ATF-4 expression involving TGF-β signaling. A central redox-responsive pathway involves NFκB signaling [56]. We next sought to determine whether this pathway-coupling PBM induced redox generation to TGF-β1/ATF-4 signaling. Preincubation with the BAY-II (NFκB) inhibitor completely eliminated ATF-4 expression from PBM, TGF-β1, or both treatments (Figure 4B). We examined the canonical Smad signaling induced by PBM and TGF-β1 and noted the robust activation of Smad 2 and 3 (Figure 4C). While SB431542 inhibition neutralized this increase as anticipated, the BAY-II inhibitor also appeared to reduce PBM-induced TGF-β signaling via Smad 2 and 3. We also observed that both ROS scavengers, NAC and Catalase, reduced the activation of TGF-β signaling following PBM treatments. These observations suggest that there is intricate crosstalk among the receptors and cytoplasmic signaling intermediates induced by PBM treatments that generates redox responses coordinated by both intracellular ATF-4/NFκB and extracellular TGF-β1 signaling.

### 3.5. NF-κB Signal Transduction Analysis

**Figure 5:**
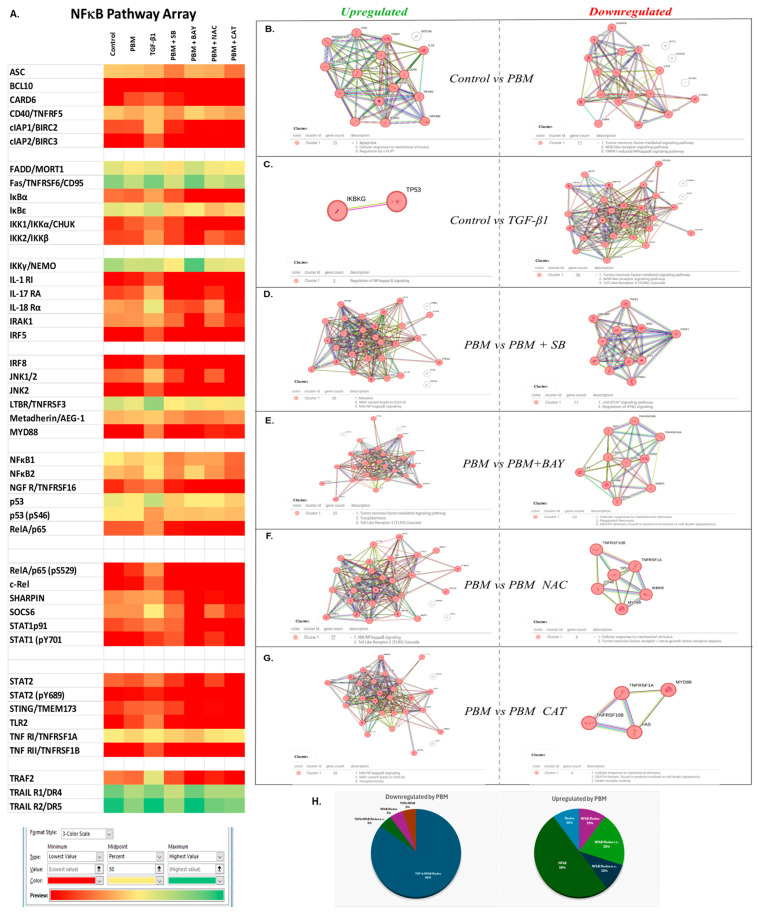
Proteomic array for NFκB signaling in oral keratinocytes. (**A**) Expression profile for protein array following PBM or TGF-β1 treatments alone or preincubated with SB431542, BAY-II, NAC, or Catalase. Cluster analyses were performed for treatment with (**B**) PBM, (**C**) TGF-β1, (**D**) SB431542, (**E**) BAY-II, (**F**) NAC, or (**G**) Catalase. (**H**) Distribution of PBM modulated candidates overlapping multiple signaling pathways. Clustering of the protein–protein interaction was performed using the *Homo sapiens* STRING database (v12.0) using the Markov Cluster Algorithm (MCL) to identify functionally coherent protein interactions.

Given the complexity of the signaling responses observed following the PBM treatments, we next performed a proteomic array of 45 members of the NF-κB pathway-specific signal transduction. We used PBM and recombinant TGF-β1 treatments alone as well as pretreatments with SB431542, BAY-II, NAC, and Catalase prior to PBM treatments that outlined discrete signatures of individual signaling interactions (Figure 5A and Supplementary Table S1). PBM treatments modulated 36 (88%) of 41 targets with the downregulation of 20 (56%), predominantly in the TNFα and NOD-like receptor signaling pathways (Figure 5B and Supplementary Figures S2A and S3). In contrast, PBM treatments upregulated 16 (44%) targets involving apoptosis, mechanotransduction, and sensing. We examined the ability of TGF-β1 treatments to modulate NFκB signaling and noted that a lower number of targets (30 of 41, 73%) were regulated, with predominantly 28 (93%) being downregulated and only 2 (7%) being upregulated (Figure 5C). The downregulated genes were also related to TNFα and NOD-like receptor signaling in the NFκB pathway, as noted for the PBM group. Comparing the two groups, a total of 18 (50%) targets appeared to overlap (Supplementary Figures S2B and S3). While 16 targets were downregulated and 1 (NEMO) was upregulated by both PBM and TGF-β1, TP53 was upregulated by PBM but downregulated by TGF-β1 (Supplementary Table S1A–C).

Pharmacologic perturbations with small molecules against TGF-β (SB431542) signaling further indicated PBM-activated TGF-β signaling. The inhibition of TGF-β signaling prior to PBM treatments upregulated 33 (97%) targets while downregulating only 1 target in the TNFα pathway (Figure 5D). The downregulated target was Stat1, which has a central role in coordinating both JAK-STAT and IFNγ signaling (Supplementary Figures S2C and S3, Supplementary Table S1D). From the prior list of 18 common targets modulated by both PBM and TGF-β1, a list of 16 PBM–TGF-β1 targets were identified, namely, CARD6, cIAP1, cIAP2, CHUK, IKK2, IL1, IL17, IRF8, MAPK8, NGFR, p65, REL, STAT2, STING1, TLR2, and TNFRSF1B. Two genes did not show changes in their expression despite the inhibitor pretreatment, namely, NEMO and STAT1, suggesting that they are independently modulated by both PBM and TGF-β1. TP53 was upregulated by PBM and downregulated by TGF-β1, but not modulated by the inhibitor, suggesting that it is regulated by alternate pathways induced by PBM treatment.

Next, 37 (90%) of the 41 targets were modulated by PBM treatment (Figure 5E). Among them, 27 (73%) targets were upregulated that clustered in the TNFα and TLR3 signaling pathway, suggesting that these were de-repressed from the PBM anti-inflammatory responses. Of these, 17 genes appear to be regulated by redox and TGF-β signaling (Supplementary Figures S2D and S3, Supplementary Table S1E). IKK2 was also specifically modulated by Catalase alone, implicating extracellular ROS. IRF8 was not modulated by NFκB inhibition but was redox- and TGF-β-signaling-dependent. In contrast, CD95 expression was upregulated following BAY and NAC, indicating that it is mediated by redox and NFκB but not TGF-β. Nine targets were upregulated following NFκB, of which five were not redox- or TGF-β-mediated, two were mediated by intracellular ROS, and one was mediated by extracellular ROS. This suggests that NF-κB activity is essential for enforcing the PBM-induced transcriptional suppression of inflammatory networks. This pattern suggests the activation of discrete stress-adaptive and regulatory nodes in parallel, coupled with the broad attenuation of inflammatory signaling rather than the engagement of a single dominant pro-inflammatory cascade.

Finally, as redox generation seems to be a fundamental intermediary step during PBM energy transfer, we examined neutralization with NAC (both intra- and extracellular) and Catalase (extracellular only) before PBM treatments. The global neutralization of PBM-generated redox with NAC upregulated 29 (83%) and downregulated 6 (17%) targets primarily associated with TNFα and TLR3 signaling (Figure 5F). Similarly, pretreatment with Catalase also downregulated 31 (89%) and upregulated 4 (11%) targets in the same pathways (Figure 5G). Among both groups, 19 PBM-downregulated targets were upregulated by both NAC and Catalase pretreatments, while 2 targets were similarly downregulated (Supplementary Figures S2E,F and S3, Supplementary Table S1F,G). Two targets, CD40 and p53, were only regulated by NAC, suggesting that PBM-generated intracellular redox regulated them. The expression of two targets, IKK2 and CD95, was reversed by Catalase pretreatment, suggesting that PBM-generated extracellular redox was primarily driving their expression. Five targets did not appear to be modulated by PBM-generated redox, namely, IL18, MORT1, NEMO, NFκB2, and TNFRSF10A.

In summary, the detailed proteomic analysis of the NFκB pathway following PBM treatments outlined the complex, interconnected signaling emanating from both intracellular and extracellular milieu. Overall, of the 36 targets modulated by PBM treatments, 17 targets were related to redox, NFκB, and TGF-β signaling, namely, CARD6, CHUK, cIAP1, cIAP2, IL1, IL17, MAPK8, MTDH, NGFR, p65, REL, SHARPIN, STAT2, STING1, TLR2, TNFRSF1B, and TP53 (Figure 5H). Two downregulated targets, STAT1 and IRF8, were both redox-mediated but appeared to be mutually exclusive for TGF-β and NFκB, respectively. All 16 upregulated targets appeared to be TGF-β-independent, while 9 were NFκB-regulated, 4 were redox-mediated, and 5 were non-redox-mediated. Among them, CD40 and p53 appeared to be intracellular-redox-regulated, while CD95 appeared to be regulated by extracellular redox generated by PBM. TNFRSF10B was redox-mediated, but not TGF-β- or NFκB-mediated. Interestingly, six targets were not regulated by TGF-β, NFκB, or redox, namely, IκBα, IκBe, LTBR, NFκB1, SOCS6, and TRAF2, which offers potential insights into non-mitochondrial PBM-activated pathways. Overall, it appears that PBM generates both predominantly redox-dependent signaling and minor redox-independent signaling initiated by PBM treatments, integrated via NFκB and TGF-β, to coordinate a delicate balance between inflammation and adaptive stress responses. These responses to PBM treatments contribute to the immediate resilience to additional stressors (like oncotherapy or hyperglycemia), while the coordinated signaling among the redox–NFκB–TGF-β pathways result in a longer-term response that drives the resolution of the inflammatory microenvironment, culminating in tissue healing and regeneration.

## 4. Discussion

**Figure 6:**
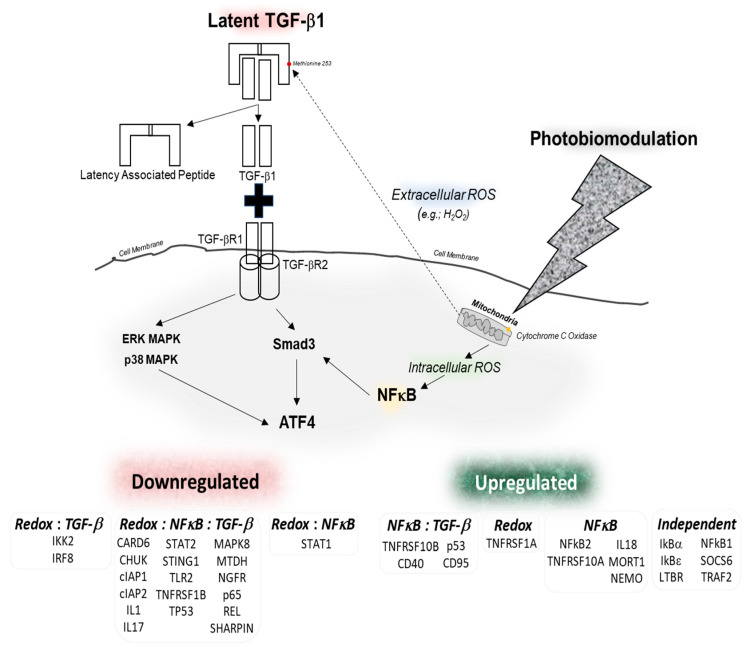
Outline of PBM-induced intracellular and extracellular signaling pathways that involve TGF-β and NFκB signaling. The hierarchy and crosstalk between the redox generated by PBM, as shown by altered mitochondrial CCO functions, activated multiple potent signaling responses that can act in a concerted manner to fine-tune the adaptive stress responses to inflammation and, further, promote resolution, leading to tissue repair and regeneration.

The utility of PBM treatments in mainstream clinical medicine has been expanding, with clinical practice guidelines in supportive cancer care recommending its routine use in mitigating several complications such as mucositis, dermatitis, and fibrosis [13,15,16,57]. A central premise for PBM applications is its potent anti-inflammatory responses to reduce damage and promote healing. This study provides a mechanistic signaling framework to assimilate four major PBM-induced responses—redox generation and TGF-β, NFκB, and ATF-4 signaling—in normal oral keratinocytes. A major observation in this study is identifying the intracellular source of PBM-induced redox generation that both contributes to NFκB intracellular signaling but also appears to diffuse extracellularly to activate latent TGF-β1 (Figure 6). The ability of PBM to activate multiple signaling pathways is unsurprising, as its pervasive nature enables transition through multiple cellular and tissue compartments. The subsequent activated latent TGF-β1 signaling appears to be fine-tuned by both canonical, specifically Smad 2, and non-canonical pathways such as Alk5, Smad3, p38, and ERK. Further, these signaling pathways appear to be modulated by concomitant intracellular redox-induced NFκB signaling, culminating in ATF-4 regulation. The role of ATF-4 in determining PBM therapeutic responses with optimal dosing appears to evoke reconstitution or removal of altered proteins initiated by integrated heat shock proteins (HSPs) and integrated stress responses (ISRs) or autophagy [25]. However, excessive PBM dosing appears to induce phototoxicity via a combination of autophagy and apoptosis, which appears to be a unique ISR signature, unlike nutritional or DNA damage. Hence, ATF-4 has been proposed as a novel biomarker to determine the upper threshold of PBM dosing [48]. The current work further strengthens the utility of these convergent, coordinated pathways, indicating that NFκB signaling represents a pivotal integration role in fine-tuning PBM–TGF-β1–ATF-4 responses.

The seminal work by Tiina Karu and Harry Whelan in the 1980s outlined the potential roles of mitochondrial cytochrome c oxidase (CCO) as a putative PBM mechanism, bringing significant credibility to the field [18,58]. Subsequent studies have further observed key roles of CCO in PBM-mediated responses, reinforcing its role in therapeutics [59,60,61,62,63]. These observations spurred careful clinical investigations that have enabled rigorous systematic reviews and meta-analyses for a broad range of ailments [64,65,66,67,68,69,70,71,72,73,74,75,76,77,78,79,80,81,82,83,84,85,86,87,88,89]. The role of CCO in transiently increasing ATP and ROS within the cell has been consistently documented in several studies [59,90,91,92,93]. The precise integration of these intracellular redox signaling pathways has remained unclear, with speculations of conventional integrated stress response (ISR) signaling converging on Nrf2-KEAP pathways [94]. Nonetheless, there are several reports questioning the universal implication of the CCO PBM mechanism that suggest there are alternate inputs or crosstalk [95,96,97,98,99,100,101]. The role of ATF-4 in specifically mediating phototoxic responses provides a novel biological signaling response within the endoplasmic reticulum that coordinates potential repair and recovery via the ISR and HSP responses [25]. This work outlines how the crosstalk between the two pathways operate in two discrete organelles: CCO in mitochondria and ATF-4 in the endoplasmic reticulum. Further, the observations in this study note that extracellular ROS occur within the cell and appear to diffuse across the cell membrane to activate latent TGF-β1 via the uniquely positioned methionine at position 253 on the latency-associated peptide [20,102]. This coordinated retrograde signaling seems central to the therapeutic clinical outcomes, evoking a resilience and repair response by PBM treatments that could explain the broad spectrum of clinical therapeutic outcomes observed.

A key role for TGF-β signaling in PBM therapeutic responses in promoting wound healing and promoting stem cells for tissue regeneration has been noted [19]. The role of TGF-β in improving tissue resilience and mediating the broad roles of the inflammatory-immune responses presents an attractive mechanistic target to ascribe PBM clinical benefits [103,104,105]. Tian F et al. (2017) demonstrated that TGF-β activates non-canonical pathways including MAPK cascades independently of SMAD signaling [54] and these pathways feedback to modulate SMAD activity. In a prior study, we noted the ability of PBM-activated TGF-β1-induced HBD-2, a potent antimicrobial agent, via both Smad and Akt signaling [106]. Our finding that Smad3 inhibition (SIS3) paradoxically increased TGF-β1-induced but decreased PBM-induced ATF-4 expression exemplifies this complex regulatory architecture, suggesting differential roles for Smad3 depending on other pathways induced by PBM treatment.

There were two major findings in the current study that provide insights into the broad range of PBM clinical responses. First, the data shows the source of redox generation by PBM within the cell that not only activates NFκB, but also diffuses outwards to activate latent TGF-β1 extracellularly. Chen et al. (2011) demonstrated that PBM treatment activates NFκB via ROS generation in mouse fibroblasts [33]. Our results extend these observations in oral keratinocytes, outlining an elaborate, coordinated signaling framework to fine-tune NFκB signaling. The pathway-specific proteomic array analysis outlines both predominant PBM-induced redox-dependent signaling and minor redox-independent signaling that are integrated via NFκB and TGF-β to coordinate a delicate balance between inflammation and adaptive stress responses. Second, the inhibition of both TGF-β (phospho-Smad 3) and ATF-4 indicates that PBM-induced, redox-activated NFκB appears to drive integrated PBM therapeutic responses, creating a feed-forward amplification or modulation loop. This could have significant implications for the ability of PBM treatments to serve as both anti-inflammatory and tissue healing therapies by evoking NFκB as an essential convergence point in the adaptive stress signaling and retrograde mitochondrial signaling network. This observation is further supported by de Farias Gabriel et al. (2019), who noted that PBM modulates NFκB expression in oral epithelial wound healing, facilitating tissue repair through coordinated proliferative rather than inflammatory gene expression [107]. ATF-4 induction by PBM represents a critical biomarker bridging oxidative signaling, growth factor activation, and endoplasmic reticulum homeostasis. Tang et al. (2024) reviewed ATF-4’s multifaceted functions in cellular stress, emphasizing that ATF-4 activation promotes either survival or death depending on stress intensity and duration [108]. Our observation that PBM-induced ATF-4 occurs alongside downregulated apoptotic signaling suggests that it mediates protective rather than terminal stress responses. This interpretation is strengthened by keratinocyte colony-forming unit assays showing enhanced proliferation of normal keratinocytes following PBM treatment, indicating that ATF-4 expression in this context supports cellular adaptation and growth rather than commitment to cell death pathways [109]. Importantly, Goswami et al. (2024) demonstrated that ATF-4 directly modulates inflammatory signaling through NFκB regulation [110], revealing bidirectional crosstalk between these transcriptional regulators.

In a recent review of the immunomodulatory effects of photobiomodulation, Al Balah et al. (2025) emphasized that PBM operates through interconnected signaling networks involving oxidative stress, mitochondrial signaling, and transcription factor activation [111]. Their analysis highlights a critical dose-dependent system: at appropriate doses, PBM exhibits anti-inflammatory properties, while higher doses trigger pro-inflammatory cascades, and oxidative stress responses serve as central mediators of PBM’s immunomodulatory effects. This work delineates how mechanistic insights can directly inform the rational design of PBM protocols. Establishing ATF-4 as a quantitative biomarker of optimal PBM exposure addresses current heterogeneity in dosimetry and outcome reporting, enabling standardized parameter selection and real-time response monitoring. The observed ATF-4 dose–response across clinically relevant irradiances supports a framework for personalized PBM treatments titrated to cellular readouts, rather than empirical treatment schedules. Mechanistically, this study outlined the central role of redox generation for PBM-induced ATF-4 via both intracellular NFκB and extracellular TGF-β1 pathways. These insights can enable precise, context-dependent PBM treatments that direct controlled pro-oxidant priming for optimal, titrated adaptive stress responses that transition to regenerative programs in wound healing or tissue engineering. Together, these data argue that PBM can concomitantly leverage endogenous growth factor and inflammatory signaling that has implications for pathophysiological context, treatment parameters, and timing, as well as clinical contraindications where appropriate.

This study has a few limitations. While three cell lines were screened, the predominant cell model investigated rigorously was the keratinocyte at an early time point (24 h). This may not capture the complexity of native oral mucosa or the full temporal kinetics of pathway activation. Future studies will incorporate organotypic 3D models or organ-on-chip platforms with multiple cell types in longitudinal time courses to examine pathway activation and downstream transcriptional programs. Future dose escalation studies could validate the pro- versus anti-inflammatory roles of redox generation as well as ATF-4 expression using pharmacologic perturbations or genetic intervention (e.g., CRISPR/Cas9 or siRNA) targeting. Translational validation in relevant rodent models will be essential to confirm efficacy and safety under optimized PBM regimens that could be employed for future human clinical studies.

## 5. Conclusions

This study outlines the complexity of PBM-treatment-induced cellular signaling that invokes redox-induced TGF-β and NFκB responses modulating adaptive stress responses via ATF-4. The concerted action of these pathways modulates cytokines, receptors, inflammatory mediators, and death pathway components. Embedding ATF-4-guided, pathway-specific endpoints in PBM preclinical and clinical studies can rationalize protocol optimization and improve reproducibility. These steps will encourage the PBM field to advance toward biomarker-driven trials with rigorous and reproducible outcomes that will ultimately result in safer and more effective interventions.

## Data Availability

The original contributions presented in this study are included in the article/Supplementary Material. Further inquiries can be directed to the corresponding author.

## Supplementary Materials

The following supporting information can be downloaded at https://www.mdpi.com/article/10.3390/cells15010088/s1, Supplementary Figure S1: Lineage analysis of PBM induced ATF-4 responses. (A) Human oral keratinocyte cell proliferation response to PBM dose escalation treatments were assessed with AlamarBlue assay (B) PBM-induced ATF-4 expression was assessed in human fibroblasts following PBM treatments assessed with western blots (C) Human cortical neurons were treated with PBM and ATF-4 expression was assessed with western blot (left) and proliferation was assessed with AlamarBlue (right). Data is presented as mean ± standard deviation where * *p* < 0.05 denotes statistically significance. Supplementary Figure S2: Proteomic array for NFκB signaling in oral keratinocytes demonstrating upregulated and downregulated genes for each group and pairwise comparisons (A) PBM treatments (B) TGF-β1 treatment and comparisons with PBM treatment group (C) PBM with preincubation SB431542 and comparison with PBM treatment group (D) PBM with preincubation BAY-II and comparison with PBM treatment group (E) PBM with preincubation NAC and comparison with PBM treatment group and (F) PBM with preincubation Catalase and comparison with PBM treatment group. Supplementary Figure S3: Tabular representation of individual candidates in the NFκB proteomic array in oral keratinocytes following PBM treatments in each condition where specific signaling pathways are blocked with small molecule inhibitors namely SB431542, BAY-II, NAC and Catalase. Supplementary Table S1: Individual group analysis of gene expression from each treatment group in the NFκB proteomic array performed in oral keratinocytes following PBM treatments with A. PBM treatments alone, B. TGF-β1 treatments, C. PBM with preincubation SB431542 and comparison with PBM treatment group, D. PBM with preincubation SB and comparison with PBM treatment group, E. PBM with preincubation BAY-II and comparison with PBM treatment group, F. PBM with preincubation NAC and comparison with PBM treatment group and G. PBM with preincubation Catalase and comparison with PBM treatment group.

## Abbreviations

The following abbreviations are used in this manuscript:

TGF-β	Transforming Growth Factor-beta
TNFα	Tumor Necrosis Factor-alpha
ATF-4	Activating Transcription Factor-4
NFκB	Nuclear Factor Kappa B
DAMP	Damage-associated Molecular Pattern
PAMP	Pathogen-associated Molecular Pattern
IκB	I-kappa-B Kinase Beta
TAK1	Transforming Growth Factor β-activated Kinase 1
NAC	N-Acetylcysteine
Stat1	Signal Transduction and Activator of Transcription 1
TP53	Tumor Protein p53
PBM	Photobiomodulation
CAT	Catalase
Smad	Mothers Against Decapentaplegic
MAPK	Mitogen-Activated Protein Kinase
CCO	Cytochrome C Oxidase
Nrf2	Nuclear Factor Erythroid 2-related Factor 2
KEAP	Kelch-like ECH-associated Protein
Alk5	Activin-Like Kinase 5
ISR	Integrated Stress Response
HSP	Heat Shock Protein
ERK	Extracellular Signal-regulated Kinase
TLR	Toll-Like Receptor
CD40	Cluster Differentiation 40
MORT1	Mediator of Receptor-Induced Toxicity-1
IL	Interleukin
STING	Stimulator of Interferon Genes
MTDH	Metadherin
CARD	CAspase Recruitment Domain
CHUK	Conserved Helix–Loop–Helix Ubiquitous Kinase
cIAP	Cellular Inhibitor of Apoptosis Proteins
