# Supplementary material for: Photobiomodulation Activates Coordinated Signaling Networks to Modulate Inflammation, Adaptive Stress, and Tissue Healing via Redox-Mediated NFκB–TGF-β1–ATF-4 Axis
Source: Cells. 2026 Jan 5;15(1):88. doi: 10.3390/cells15010088 (PMC12785302; doi:10.3390/cells15010088)
Supplement: Supplementary file 1 [file cells-15-00088-s001.zip › cells-4079346-supplementary.pdf]

Supplementary Figure S1

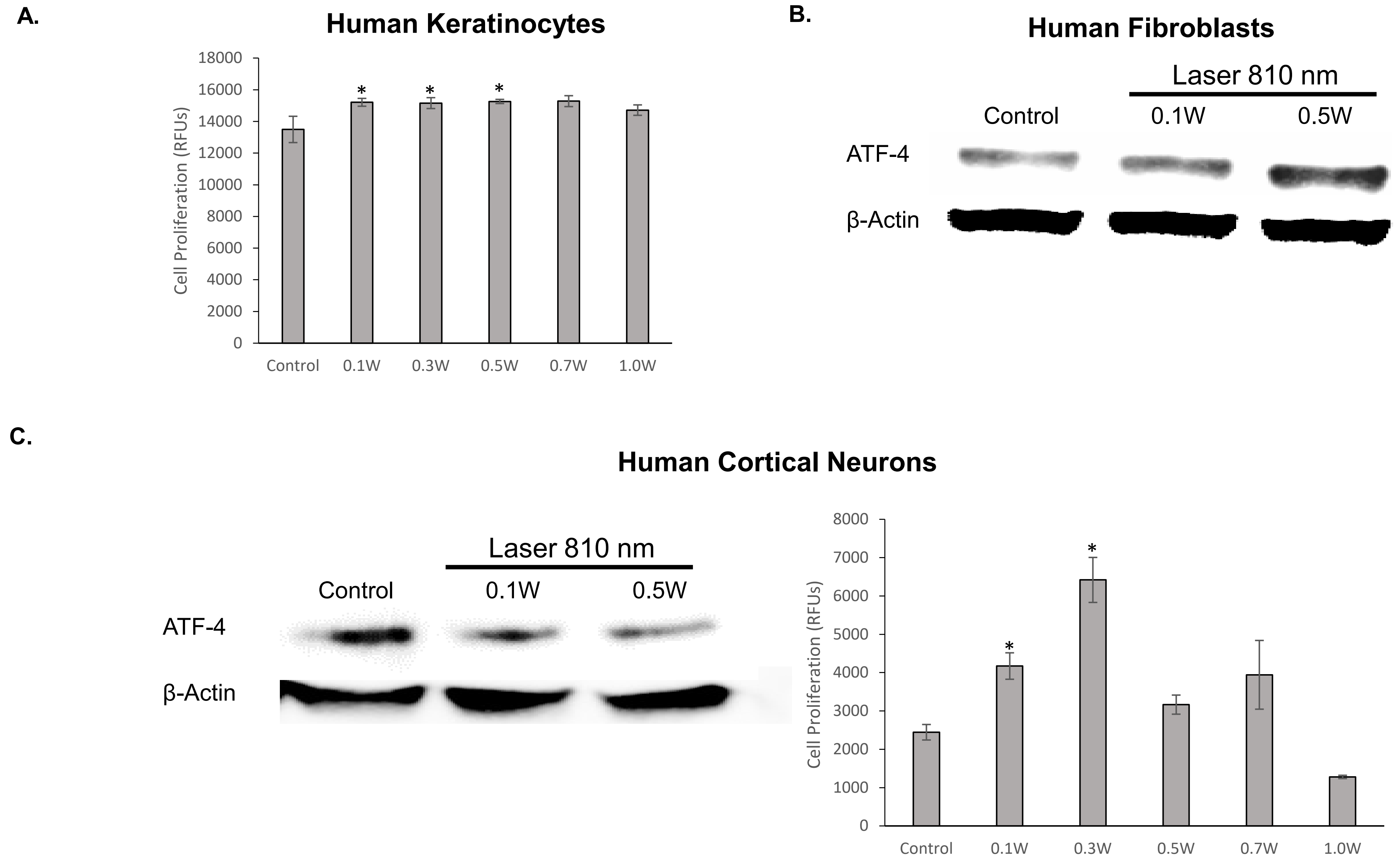

Lineage analysis of PBM induced ATF-4 responses. (**A**) Human oral keratinocyte cell proliferation response to PBM dose escalation treatments were assessed with AlamarBlue assay (**B**) PBM-induced ATF-4 expression was assessed in human fibroblasts following PBM treatments assessed with western blots (**C**) Human cortical neurons were treated with PBM and ATF-4 expression was assessed with western blot (left) and proliferation was assessed with AlamarBlue (right). Data is presented as mean $\pm$ standard deviation where *$p < 0.05$ denotes statistically significance.

Supplementary Figure S2

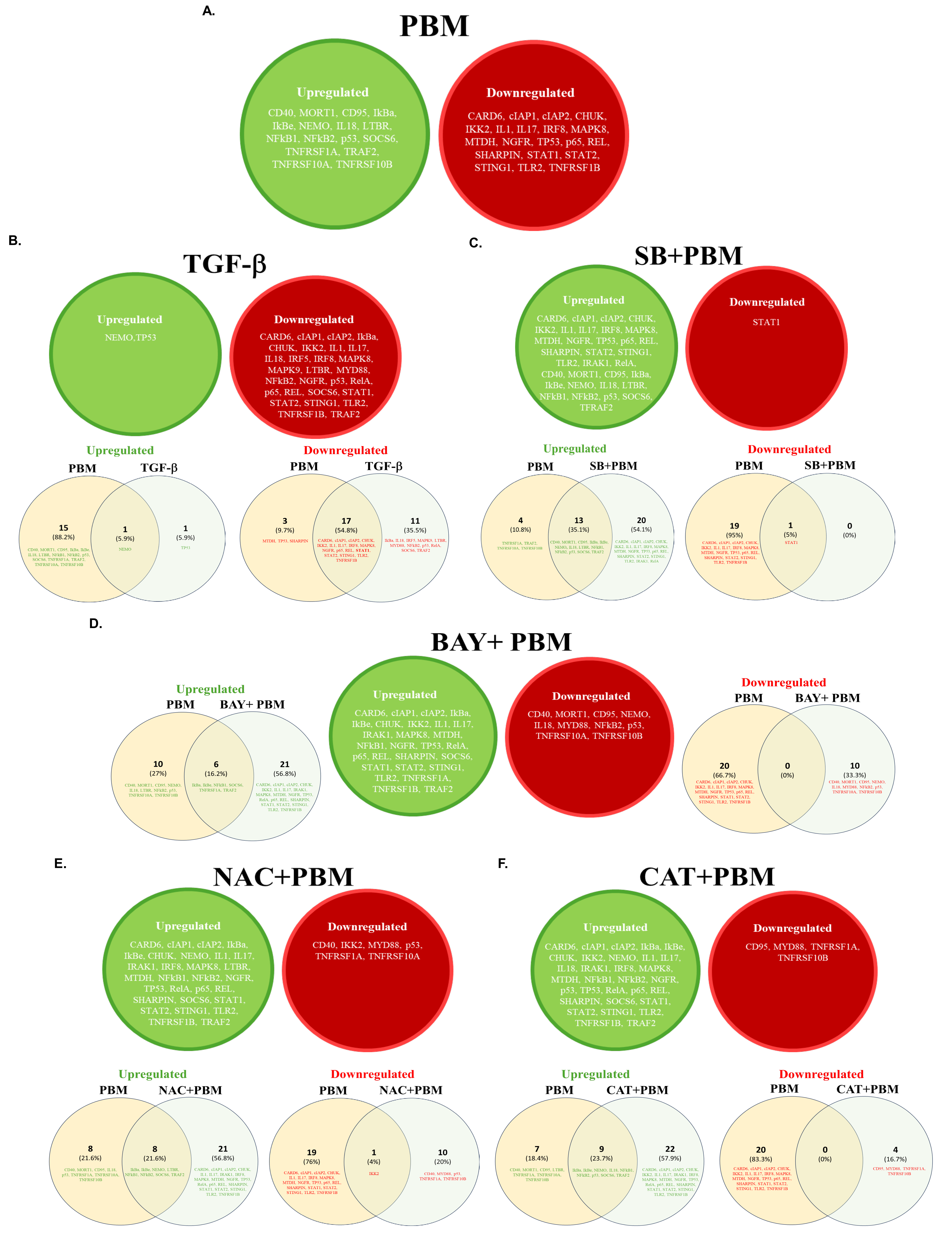

Proteomic array for NFκB signaling in oral keratinocytes demonstrating upregulatred and downregulated genes for each group and pairwise comparisons (**A.**) PBM treatments (**B.**) TGF-β1 treatment and comparisons with PBM treatment group (**C.**) PBM with preincubation SB431542 and comparison with PBM treatment group (**D.**) PBM with preincubation BAY-II and comparison with PBM treatment group (**E.**) PBM with preincubation NAC and comparison with PBM treatment group and (**F.**) PBM with preincubation Catalase and comparison with PBM treatment group

Supplementary Figure S3

PBM Regulated	SB431542	BAY II	NAC	CAT
CARD6	■	■	■	■
CD40		■	■	
CD95		■		■
CHUK	■	■	■	■
cIAP1	■	■	■	■
cIAP2	■	■	■	■
IkBa				
IkBe				
IKK2	■	■		■
IL1	■	■	■	■
IL17	■	■	■	■
IL18		■		
IRAK1				
IRF5				
IRF8	■		■	■
LTBR				
MAPK8	■	■	■	■
MAPK9				
MORT1		■		
MTDH	■	■	■	■
MYD88		■	■	■
NEMO		■		
NFkB1				
NFkB2		■		
NGFR	■	■	■	■
p53		■	■	
p65	■	■	■	■
REL	■	■	■	■
RelA				
SHARPIN	■	■	■	■
SOCS6				
STAT1		■	■	■
STAT2	■	■	■	■
STING1	■	■	■	■
TLR2	■	■	■	■
TNFRSF10A		■		
TNFRSF10B		■	■	■
TNFRSF1A			■	■
TNFRSF1B	■	■	■	■
TP53	■	■	■	■
TRAF2				

Tabular representation of individual candidates in the NFκB proteomic array in oral keratinocytes following PBM treatments in each condition where specific signaling pathways are blocked with small molecule inhibitors namely SB431542, BAY-II, NAC and Catalase.

Supplementary Table S1

Inducers

A.

PBM (Compared to Control)

Upregulated	Downregulated	No Change
CD40	CARD6	IRAK1
CD95	CHUK	IRF5
IkBa	cIAP1	MAPK9
IkBe	cIAP2	MYD88
IL18	IKK2	RelA
LTBR	IL1	
MORT1	IL17	
NEMO	IRF8	
NFkB1	MAPK8	
NFkB2	MTDH	
p53	NGFR	
SOCS6	p65	
TNFRSF10A	REL	
TNFRSF10B	SHARPIN	
TNFRSF1A	STAT1	
TRAF2	STAT2	
	STING1	
	TLR2	
	TNFRSF1B	
	TP53	

B.

TGF-β (compared to Control)

Upregulated	Downregulated	No Change
NEMO	CARD6	CD40
TP53	CHUK	CD95
	cIAP1	IkBe
	cIAP2	IRAK1
	IkBa	MORT1
	IKK2	MTDH
	IL1	NFkB1
	IL17	SHARPIN
	IL18	TNFRSF10A
	IRF5	TNFRSF10B
	IRF8	TNFRSF1A
	LTBR	
	MAPK8	
	MAPK9	
	MYD88	
	NFkB2	
	NGFR	
	p53	
	p65	
	REL	
	RelA	
	SOCS6	
	STAT1	
	STAT2	
	STING1	
	TLR2	
	TNFRSF1B	
	TRAF2	

C.

PBM Only	PBM:TGFB		TGF-b Only
MTDH	CARD6	CARD6	IKK2
SHARPIN	CHUK	CHUK	IRF8
TP53	cIAP1	cIAP1	
	cIAP2	cIAP2	
	IL1	IL1	
	IL17	IL17	
	MAPK8	MAPK8	
	NGFR	NGFR	
	p65	p65	
	REL	REL	
	STAT2	STAT2	
	STING1	STING1	
	TLR2	TLR2	
	TNFRSF1B	TNFRSF1B	
	TP53	TP53	
	NEMO	NEMO	

Individual group analysis of gene expression from each treatment group in the NFκB proteomic array performed in oral keratinocytes following PBM treatments with **A.** PBM treatments alone, **B**. TGF-β1 treatments (*continued*)

Supplementary Table S1

Repressors

D.

SB+PBM (compared to PBM)		
**Upregulated**	**Downregulated**	**No Change**
CARD6	STAT1	IRF5
CD40		MAPK9
CD95		MYD88
CHUK		TNFRSF10A
cIAP1		TNFRSF10B
cIAP2		TNFRSF1A
IkBa		TNFRSF1B
IkBe		
IKK2		
IL1		
IL17		
IL18		
IRAK1		
IRF8		
LTBR		
MAPK8		
MORT1		
MTDH		
NEMO		
NFkB1		
NFkB2		
NGFR		
p53		
p65		
REL		
RelA		
SHARPIN		
SOCS6		
STAT2		
STING1		
TLR2		
TP53		
TRAF2		

E.

BAY+PBM (compared to PBM)		
**Upregulated**	**Downregulated**	**No Change**
CARD6	CD40	IRF5
CHUK	CD95	IRF8
cIAP1	IL18	LTBR
cIAP2	MORT1	MAPK9
IkBa	MYD88	
IkBe	NEMO	
IKK2	NFkB2	
IL1	p53	
IL17	TNFRSF10A	
IRAK1	TNFRSF10B	
MAPK8		
MTDH		
NFkB1		
NGFR		
p65		
REL		
RelA		
SHARPIN		
SOCS6		
STAT1		
STAT2		
STING1		
TLR2		
TNFRSF1A		
TNFRSF1B		
TP53		
TRAF2		

F.

NAC+PBM (compared to PBM)		
**Upregulated**	**Downregulated**	**No Change**
CARD6	CD40	CD95
CHUK	IKK2	IL18
cIAP1	MYD88	IRF5
cIAP2	p53	MAPK9
IkBa	TNFRSF10B	MORT1
IkBe	TNFRSF1A	TNFRSF10A
IL1		
IL17		
IRAK1		
IRF8		
LTBR		
MAPK8		
MTDH		
NEMO		
NFkB1		
NFkB2		
NGFR		
p65		
REL		
RelA		
SHARPIN		
SOCS6		
STAT1		
STAT2		
STING1		
TLR2		
TNFRSF1B		
TP53		
TRAF2		

G.

CAT+PBM (compared to PBM)		
**Upregulated**	**Downregulated**	**No Change**
CARD6	CD95	CD40
CHUK	MYD88	IRF5
cIAP1	TNFRSF10B	LTBR
cIAP2	TNFRSF1A	MAPK9
IkBa		MORT1
IkBe		TNFRSF10A
IKK2		
IL1		
IL17		
IL18		
IRAK1		
IRF8		
MAPK8		
MTDH		
NEMO		
NFkB1		
NFkB2		
NGFR		
p53		
p65		
REL		
RelA		
SHARPIN		
SOCS6		
STAT1		
STAT2		
STING1		
TLR2		
TNFRSF1B		
TP53		
TRAF2		

(*Continued*) Individual group analysis of gene expression from each treatment group in the NFκB proteomic array performed in oral keratinocytes following PBM treatments with **C.** PBM with preincubation SB431542 and comparison with PBM treatment group, **D.** PBM with preincubation SB and comparison with PBM treatment group **E.** PBM with preincubation BAY-II and comparison with PBM treatment group **F.** PBM with preincubation NAC and comparison with PBM treatment group and **G.** PBM with preincubation Catalase and comparison with PBM treatment group
